# Persistence of Pro-Arrhythmic Spatio-Temporal Calcium Patterns in Atrial Myocytes: A Computational Study of Ping Waves

**DOI:** 10.3389/fphys.2012.00279

**Published:** 2012-07-20

**Authors:** Rüdiger Thul, Stephen Coombes, Martin D. Bootman

**Affiliations:** ^1^School of Mathematical Sciences, University of NottinghamNottingham, UK; ^2^Laboratory of Signaling and Cell Fate, The Babraham InstituteCambridge, UK; ^3^Department of Life, Health and Chemical Sciences, The Open UniversityMilton Keynes, UK

**Keywords:** atrial myocyte, Ca^2+^ signaling, arrhythmia

## Abstract

Clusters of ryanodine receptors within atrial myocytes are confined to spatially separated layers. In many species, these layers are not juxtaposed by invaginations of the plasma membrane (transverse tubules; ‘T-tubules’), so that calcium-induced-calcium signals rely on centripetal propagation rather than voltage-synchronized channel openings to invade the interior of the cell and trigger contraction. The combination of this specific cellular geometry and dynamics of calcium release can lead to novel autonomous spatio-temporal calcium waves, and in particular ping waves. These are waves of calcium release activity that spread as counter-rotating sectors of elevated calcium within a single layer of ryanodine receptors, and can seed further longitudinal calcium waves. Here we show, using a computational model, that these calcium waves can dominate the response of a cell to electrical pacing and hence are pro-arrhythmic. This highlights the importance of modeling internal cellular structures when investigating mechanisms of cardiac dysfunction such as atrial arrhythmia.

## 1 Introduction

The most common form of cardiac dysrhythmia in humans is “atrial fibrillation.” This pathology arises when electrical impulses occur spontaneously, and with high frequency, from sites around the atria (typically 350 discharges per minute compared to the normal sinoatrial rhythm of 60–80 beats per minute; Dobrev, 2010). Due to these irregular electrical discharges, the atria do not display coordinated contractions required to propel blood into the ventricles. Consequently, the blood pumping capacity of the heart can be reduced by up to a third (Alpert et al., 1988). Given that ventricles can refill substantially without atrial participation, atrial fibrillation is typically not immediately life threatening. However, the loss of atrial contraction can be debilitating when greater output from the heart is required, for example during physical exertion. A further complication associated with atrial fibrillation is thromboembolism due to the stagnation of blood within the atrial chambers. With respect to human health, it is established that the incidence of atrial fibrillation increases with age, and ∼15% of strokes occur in people with atrial fibrillation. It is therefore clear that coordinated atrial function is very important. Substantial evidence points to dysregulation of calcium (Ca^2+^) signaling as being a causal factor in genesis and maintenance of atrial fibrillation (Hove-Madsen et al., 2004; Dobrev and Nattel, 2008; Yeh et al., 2008; Wakili et al., 2010; Greiser et al., 2011).

Ca^2+^ is the key regulator of heart contraction (Bers, 2008). Each heart beat is associated with fluxes of Ca^2+^ across the cardiac myocyte plasma membrane, the sarcolemma, and from the internal Ca^2+^ store, the sarcoplasmic reticulum (SR). Cardiac excitation-contraction coupling (EC-coupling) is initiated by an action potential that sweeps from the sinoatrial node, the pacemaking region of the heart. When the depolarizing action potential reaches individual myocytes it causes depolarization of their sarcolemma leading to activation of L-type voltage-operated Ca^2+^ channels (VOCs), and consequently a brief influx of Ca^2+^. The Ca^2+^ influx through VOCs provides a trigger signal to provoke more substantial Ca^2+^ release from closely apposed ryanodine receptor (RyR) clusters on the SR, by a process known as Ca^2+^-induced Ca^2+^ release (CICR; Callewaert, 1992; Roderick et al., 2003). Activation of RyRs leads to the generation of elementary Ca^2+^ signals known as a Ca^2+^ sparks. These microscopic signals essentially reflect the simultaneous activation of a cluster of RyRs by CICR (Cheng et al., 1993; Cannell et al., 1995; Rios, 2005). The spatial overlap and temporal summation of signals from multiple Ca^2+^ spark sites underlies the rapid homogenous Ca^2+^ transients that trigger coordinated ventricular myocyte contraction (Cannell et al., 1995; Bootman et al., 2001; Guatimosim et al., 2002).

EC-coupling in atrial myocytes is substantially different from that in ventricular cells (Bootman et al., 2006). In most species, atrial cells lack the T-tubule invaginations of the sarcolemma found in ventricular myocytes (Brette and Orchard, 2003), and therefore express VOCs only on the sarcolemma surrounding the cells (Bootman et al., 2011). The distribution of RyRs in atrial cells is similar to that in ventricular myocytes, but with the important exception that only a small fraction of the RyRs (the junctional RyRs) are positioned to respond to the opening of the VOCs (Carl et al., 1995; Mackenzie et al., 2001). Ca^2+^ signals in atrial myocytes therefore originate around the periphery of the cells and are locally amplified by the junctional RyRs.

Under control conditions, this peripheral Ca^2+^ signal does not propagate fully, or at all, into the center of an atrial cell. This means that at the peak of the response, substantial Ca^2+^ gradients can be observed (Berlin, 1995; Mackenzie et al., 2001; Woo et al., 2002; Sheehan and Blatter, 2003). However, in addition to the junctional RyRs, atrial myocytes have a regular 3-dimensional lattice of “non-junctional” RyR clusters, which pervade the entire cytoplasmic compartment (Chen-Izu et al., 2006). It could be expected that the subsarcolemmal Ca^2+^ signal arising from the junctional RyRs would be sensed and amplified by the non-junctional RyRs via CICR. In this way, the trigger Ca^2+^ signal in the cell periphery could lead to centripetal propagation of a Ca^2+^ wave and complete engulfment of the cell. However, in the absence of inotropic stimulation, the non-junctional RyRs in the center of atrial myocytes are largely non-responsive. This is due to a 2 μm gap in expression of RyRs between the junctional and non-junctional RyRs and cellular buffering mechanisms that inhibit inward propagation of the Ca^2+^ signal (Mackenzie et al., 2004). To stimulate contraction, the Ca^2+^ signal has to overcome the buffers and invade the cell center where the bulk of myofilaments exists (Bootman et al., 2006).

To explore the factors controlling Ca^2+^ homeostasis and signaling in atrial myocytes, we have developed a mathematical model based on the established geometry of the cells (Thul et al., 2012). The model incorporates multiple discrete Ca^2+^ release sites within the volume of the *in-silico* myocyte that have the same defined spacing as in a real atrial cell. The movement of Ca^2+^ within the atrial myocyte model, and the triggering of Ca^2+^ release is based on realistic terms for Ca^2+^ fluxes and diffusion. We previously validated the model by recapitulating the centripetal propagation of Ca^2+^ waves observed under physiological stimulation conditions. Moreover, we examined the impact of inotropic stimulation on Ca^2+^ movement, and were able to unravel the contributions of factors such as SR Ca^2+^ load, RyR sensitization, and increased VOC activity to spatio-temporal Ca^2+^ wave propagation (Thul et al., 2012). In addition to replicating physiological patterns of Ca^2+^ movement, the model demonstrates the potential for developing spontaneous Ca^2+^ signals, which could have a pro-arrhythmic capacity in the heart. One such arrhythmic Ca^2+^ pattern that emerges is a self-sustaining, rotating Ca^2+^ wave that we termed a “ping wave.” Ping waves are particularly interesting in that they require conditions of relatively low Ca^2+^ flux – akin to the situation in heart failure where RyR and Ca^2+^ pump expression significantly declines (Goldhaber and Bridge, 2009). Ping waves are dependent on perpetual Ca^2+^ release within a discrete region of an atrial myocyte, but are ultimately able to trigger repetitive Ca^2+^ waves throughout the volume of the cell. We believe that ping waves may represent a hitherto unseen form of spontaneous Ca^2+^ release that is unavoidable under certain stimulation conditions due to the geometry of atrial myocytes. As yet, ping waves have not been visualized using experimental techniques because they are beyond the scope of current imaging technology. In this study, we present further characterization of ping waves. We demonstrate their robustness in the face of electrical pacing, and their sensitivity to the refractory state of the underlying RyRs.

## 2 Materials and Methods

We employ a recently developed three-dimensional model of an atrial myocyte (Thul et al., 2012). The spatio-temporal dynamics of the cytosolic Ca^2+^ concentration *c*(**r**, *t*) is governed by

(1) $$\frac{\partial c}{\partial t}=D\Delta c-\frac{c}{\tau }+\underset{n\in \Gamma }{\sum }\underset{m\in \mathbb{N}}{\sum }\delta \left(r-r_{n}\right)\eta \left(t-T_{n}^{m}\right)\;,$$

where *D* denotes the effective cytosolic diffusion coefficient and τ represents the sequestration time scale of the SERCA pumps. Ca^2+^ release from the SR into the cytosol occurs at discrete positions **r***_n_*, n ∈ Γ. The time at which the *n*th release site opens for the *m*th time is given by $T_{n}^{m}$ and is implicitly defined by

(2) $$T_{n}^{m}=\text{inf}\left\{t\;|\;c\left(r_{n},t\right)>c_{\text{th}},\;T_{n}^{m}>T_{n}^{m-1}+t_{\text{ref}}\right\}.$$

**Figure 1:**
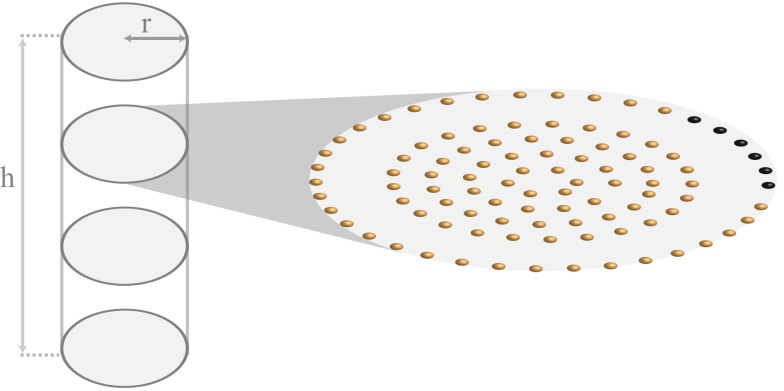
**Schematic representation of the cylindrical geometry of the atrial myocyte model**. The cylinder has a height of *h *= 100.4 μm and a radius of *r *= 6 μm. A z-plane transverses the cylinder every 2 μm along the longitudinal axis. For illustrative purposes, only 4 of the 51 z-planes are displayed. The zoom-in shows the arrangement of the RyRs within a single z-plane. Each small sphere corresponds to a cluster of RyRs. The spacing between RyRs along a ring and between rings of non-junctional Ca^2+^ release is 1 μm. The gap between junctional and non-junctional RyRs is 2 μm. Please refer to text for the color-coding of the peripheral the RyRs.

Ca^2+^ release occurs when the cytosolic Ca^2+^ concentration is above a threshold value *c*_th_, and there is at least a refractory period *t*_ref_ between consecutive release events. The exact shape of the Ca^2+^ release current is captured by the function η,which we here take as piecewise constant, i.e., η(*t*) = σ*H*(*t*)*H*(*t*_rel_ − *t*). The Ca^2+^ release strength is measured by σ, and the Heaviside function *H*, which is zero for negative arguments and 1 otherwise, ensures that release occurs over a period of *t*_rel_. We solve equation (1) in a cylinder of length *h *= 100.4 μm and radius of *a *= 6 μm, consistent with the actual shape and size of an atrial myocyte (Bootman et al., 2011). Ca^2+^ release is restricted to planes that are perpendicular to the longitudinal axis of the cylinder and are separated by ∼2 μm. Within each of these so-called z-planes, RyRs are arranged in a circular pattern as shown in Figure 1. The distance between RyRs within a single ring and between the four inner rings is ∼1 μm. The gap between the peripheral RyRs and the non-junctional Ca^2+^ release sites measures ∼2 μm and corresponds to the experimentally observed lack of expression of RyRs (Bootman et al., 2011).

To mimic periodic pacing we elevate the Ca^2+^ concentration of a given fraction of peripheral RyRs above threshold at each pacing event. We keep this initial fraction constant from pacing event to pacing event, but we randomize the position of the activated peripheral Ca^2+^ release sites with each pacing event.

We refer the reader to section 4 for a more detailed discussion of the model. The default parameter values for all simulations are listed in Table 1.

**Table 1:** **Default parameter values for simulations**.

Parameter	Value	Unit
Height of cylinder *l*	100.4	μm
Radius of cylinder *R*	6	μm
Diffusion coefficient of Ca^2+^ *D*	30	μm^2^ s^−1^
Pump strength τ	0.4	s
Release time *t*_rel_	0.05	s
Refractory time *t*_ref_	0.5	s
Threshold Ca^2+^ concentration *c*_th_	0.15	μM
Radial discretization d*r*	0.1	μm
Angular discretization dθ	0.0838	
Axial discretization d*z*	0.1	μm
Temporal discretization d*t*	0.01	s

## 3 Results

**Figure 2:**
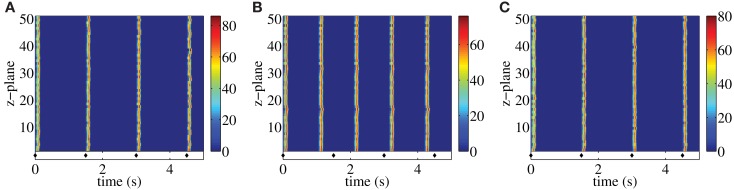
**Paced myocyte with an initial fraction of 0.2 for different combinations of release strength and refractory period: σ = 15 μM μm^3^/s, *t*_ref_ = 1.4 s (A), σ = 15 μM μm^3^/s, *t*_ref_ = 1 s (B), σ = 13 μM μm^3^/s, *t*_ref_ = 1 s (C)**. Each stimulation is indicated by a diamond symbol. Warm colors indicate substantial Ca^2+^ release site activity, while cool colors correspond to lesser Ca^2+^ release activity. Other parameter values are as in Table 1.

Following each systolic Ca^2+^ transient RyRs are refractory for a period of time (DelPrincipe et al., 1999; Terentyev et al., 2002; Szentesi et al., 2004; Sobie et al., 2005; Ramay et al., 2011; Kornyeyev et al., 2012). This refractoriness is believed to be essential for myocytes to reset their EC-coupling machinery in time for the next heart beat. Consistent with this notion, our previous study (Thul et al., 2012) identified the refractory period of RyRs as a key determinant of the fidelity of centripetal Ca^2+^ waves or pro-arrhythmic Ca^2+^ signals. Essentially, the duration of the refractory period determines when RyRs are available for activation following a previous stimulation. If the refractory period is relatively short, then a cell may not have sufficient time to reduce cytosolic Ca^2+^, and RyRs can be spontaneously activated as soon as they emerge from their refractory state. This situation would be pro-arrhythmic. Whilst the refractory period is vital, it is co-dependent on other factors that determine the sensitivity of RyRs to Ca^2+^ and the amount of Ca^2+^ released. This is exemplified in Figure 2, which illustrates the interplay between refractory period and release strength in controlling the fidelity of Ca^2+^ signals in a paced atrial myocyte. Figure 2A shows a paced model atrial myocyte under control conditions. Each pacing event is indicated by a diamond symbol and initiates a centripetal Ca^2+^ wave, akin to those observed in contracting myocytes (Bootman et al., 2011). The centripetal Ca^2+^ waves are evident as vertical lines in the top panel that align with the diamonds below. For the parameters used in Figure 2A, the refractory period is sufficiently long that the residual Ca^2+^ concentration falls below the threshold value for triggering Ca^2+^ liberation when the RyRs exit the refractory period. Hence, cells require repetitive pacing to trigger subsequent Ca^2+^ waves. Shortening the refractory period allows RyRs to emerge from their refractory state whilst the ambient Ca^2+^ concentration is sufficiently high to cause their spontaneous activation. Figure 2B shows such a situation – after the first pacing event autonomous Ca^2+^ waves are triggered and become the sole subsequent activity (note the mismatch between the diamond symbols and the Ca^2+^ waves). The initiation of the unpaced Ca^2+^ waves is due to the supercritical value of the residual Ca^2+^ concentration when the RyRs emerge from being refractory. Keeping the same value of the refractory period as in Figure 2B, we can restore externally triggered Ca^2+^ waves by lowering the release strength as illustrated in Figure 2C. These results demonstrate that for a given release strength, there is a minimum refractory period below which autonomous Ca^2+^ waves occur. The larger the release strength, the longer is the time window for initiating stimulus-independent Ca^2+^ waves.

**Figure 3:**
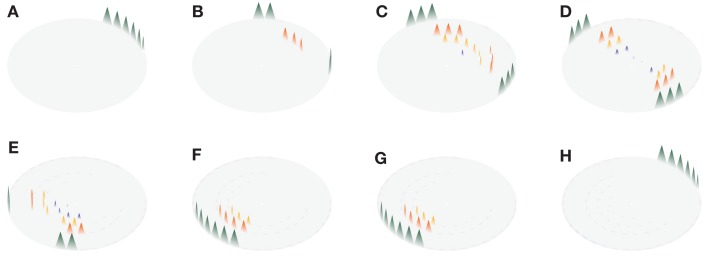
**Ping wave with a refractory period *t*_ref_ = 0.32 s and release strength σ = 8.5 μM μm^3^/s shown at different time points**. The eight panels cover one revolution of the ping wave. Time increases from **(A–H)** as *t* = 0.02 s **(A)**, 0.09 s **(B)**, 0.14 s **(C)**, 0.20 s **(D)**, 0.29 s **(E)**, 0.33 s **(F)** 0.36 s **(G)** and 0.38 s **(F)**. The triangular shape of the Ca^2+^ concentration profiles is for illustrative purposes only. Other parameter values as in Table 1.

Another manifestation of self-sustaining, spontaneous Ca^2+^ waves that derives from the specific geometry of atrial myocytes occurs within a single z-plane under appropriate conditions of refractory period and Ca^2+^ release strength. Specifically, the limited recruitment of a few RyR clusters triggers a perpetual, rotating Ca^2+^ ping wave within a z-plane (Thul et al., 2012). An example of such a ping wave is depicted in Figure 3. In the example shown, the ping wave initiates from 6 adjacent RyR clusters (Figure 3A). As time progresses two counter-rotating sectors of Ca^2+^ release activity emerge, sweeping from one side of the z-plane to the other (Figures 3B–G). The time it takes for one of the sectors to traverse half the z-plane equals the sum of the release duration and the refractory period (0.37 s), so that Ca^2+^ release reinitiates at one side of the z-plane as soon as the sectors reach the opposite side (cf. Figures 3A,H at *t *= 0.02 s and *t *= 0.38 s, respectively).

**Figure 4:**
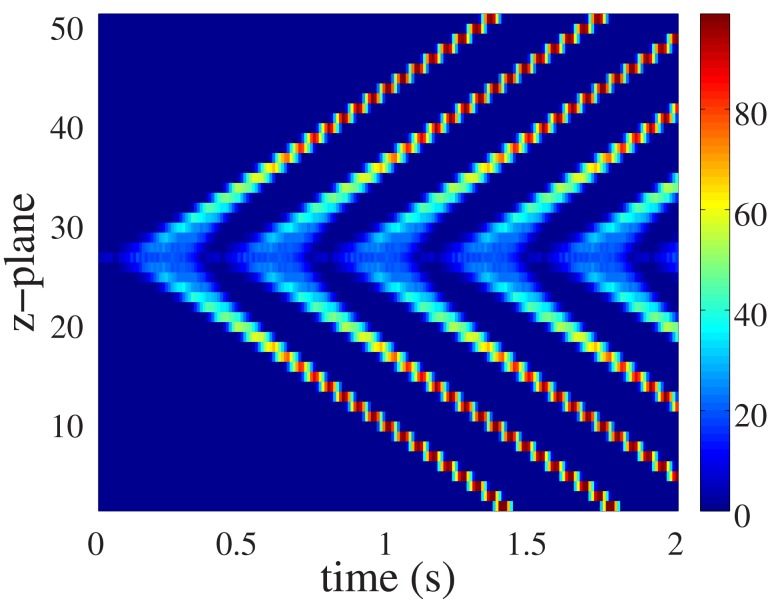
**Ping wave and longitudinal Ca^2+^ waves for the parameter values in Figure 3**. The ping wave is triggered by initially elevating the Ca^2+^ concentration above threshold at the six release sites colored black in Figure 1 in the central z-plane. Warm colors indicate substantial Ca^2+^ release site activity, while cool colors correspond to lesser Ca^2+^ release activity.

Although ping waves occur within a single z-plane, their influence is not restricted to that domain of the cell. Rather, they have the capacity to trigger longitudinal Ca^2+^ waves that pervade the entire cellular volume. Figure 4 illustrates how a ping wave perpetually rotating within a single plane can seed repetitive longitudinal Ca^2+^ waves. The ping wave is clearly visible as the light bluish ribbon running along the center of the Figure. Emanating from the ping wave activity are diagonal rays of elevated Ca^2+^ activity that correspond to longitudinal Ca^2+^ waves. The only stimulus required for all the Ca^2+^ activity evident in Figure 4 was the initial triggering of 6 peripheral RyR clusters (as in Figure 3A). All subsequent activity resulted from that initial spatially-restricted RyR activation. In a myocyte where all RyRs have the same refractory period *t*_ref_, longitudinal Ca^2+^ waves also exhibit periods with duration *t*_ref_. The perpetual, ping wave Ca^2+^ release activity only occurs in the central z-plane. In all other z-planes, there is a clear quiescence phase between consecutive longitudinal waves. In the remainder of this paper, we will address the robustness of ping waves, especially with respect to physiologically relevant pacing conditions.

**Figure 5:**
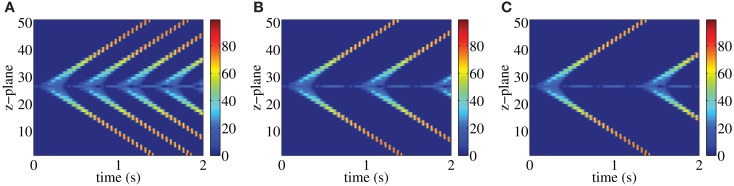
**Ping wave initiated as in Figure 4 with a mismatch in the refractory period**. The refractory period in the central z-plane is 0.34 s, while *t*_ref_ = 0.4 s **(A)**, 0.8 s **(B)**, and 1.2 s **(C)** in the rest of the cell. Warm colors indicate substantial Ca^2+^ release site activity, while cool colors correspond to lesser Ca^2+^ release activity. Other parameter values as in Table 1 and σ = 8.5 μM μm^3^/s.

In the first step, we examined how changing the refractory period of RyRs affected either the existence of ping waves, or the seeding of longitudinal Ca^2+^ waves. With this aim, we increased the refractory period at all z-planes except the one where we triggered the ping wave. Figure 5 demonstrates that this manipulation does not prevent the rotation of ping waves or the seeding of longitudinal Ca^2+^ waves. As soon as the RyRs outside the central z-plane exit the refractory period, the ping wave triggers the longitudinal Ca^2+^ wave. Increasing the refractory period further (Figures 5B,C) yields similar results. Essentially, ping waves are a perpetual trigger for CICR. They can persist in isolation until RyRs in neighboring z-planes recover from being refractory. The small mismatch of the refractory period in the z-plane of the ping wave and the rest of the myocyte in Figure 5A leads to longitudinal Ca^2+^ waves that begin at different phases of the ping wave, indicating that ping waves can seed longitudinal Ca^2+^ waves at any point of their rotation.

**Figure 6:**
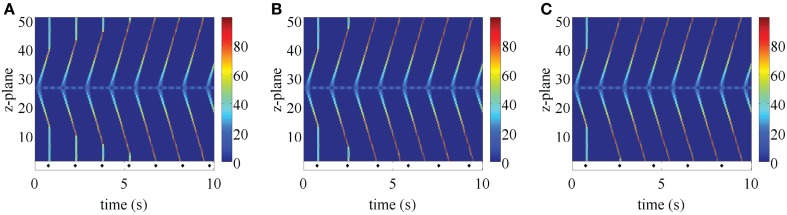
**Paced ping wave initiated as in Figure 4 for a pacing period of *t*_p_ = 1.5 s (A), 1.7 s (B), and 1.9 s (C)**. Each stimulation is indicated by a diamond symbol. The first pacing pulse is delivered at 0.75 s. The refractory period in the central z-plane is 0.34 s, while *t*_ref_ = 1.3 s in the rest of the cell. Warm colors indicate substantial Ca^2+^ release site activity, while cool colors correspond to lesser Ca^2+^ release activity. Other parameter values as in Table 1 and σ = 8.5 μM μm^3^/s, initial fraction 1.0.

The robust initiation of longitudinal Ca^2+^ waves makes ping waves a prime candidate to induce arrhythmias. However, the results in Figures 3–5 considered a situation where cells are electrically silent. In the context of the heart, they would be receiving electrical pacing from the sinoatrial node. As we have demonstrated (Bootman et al., 2011; Figure 2), physiological pacing provokes centripetal Ca^2+^ waves to drive contraction. We therefore examined what would happen to pacing-evoked centripetal Ca^2+^ waves if they were triggered in a myocyte that had an established ping wave. We use the same initial conditions as in Figure 5 to generate a ping wave, and after a fixed delay trigger the activation of the peripheral RyRs to simulate electrical pacing. The outcomes of beginning pacing 1.5, 1.7, and 1.9 s after initiating a ping wave are shown in Figure 6. The responses include a complex mixture of ping waves, longitudinal Ca^2+^ waves and centripetal Ca^2+^ waves. The ping wave is evident as the bluish band running through the middle of the plots. The longitudinal Ca^2+^ waves run diagonally, whereas the pacing-evoked centripetal Ca^2+^ waves are vertical. Unlike the pattern shown in Figure 2, the centripetal Ca^2+^ waves do not invade the entirety of the cell. When a longitudinal Ca^2+^ wave and centripetal Ca^2+^ wave meet they annihilate each other, and Ca^2+^ release activity is abruptly terminated. However, since the ping wave is still active, further longitudinal Ca^2+^ waves are triggered. The plots in Figure 6 show a progressive change in the response of the cell as the longitudinal Ca^2+^ waves become progressively more dominant over the centripetal Ca^2+^ waves. Since the pacing period is longer than the refractory period, the longitudinal Ca^2+^ wave can travel further through the cell before the next pacing stimulus is applied. Therefore the longitudinal Ca^2+^ wave and centripetal Ca^2+^ wave meet and annihilate closer to the edge of the cell. This pattern keeps on repeating, until the longitudinal Ca^2+^ wave eventually reaches the top and the bottom of the myocyte, rendering all future pacing events ineffective. Increasing the pacing period *t*_p_ does not change this behavior. A larger value of *t*_p_ only results in a smaller number of centripetal Ca^2+^ waves before a fully developed longitudinal Ca^2+^ wave occurs. For example, in Figure 6A, there are 4 evident centripetal Ca^2+^ waves, while only two such waves occur in Figure 6B compared to one in Figure 6C. Note that shortening the initial delay yields qualitatively similar results (data not shown). At the core of these outcomes is the ping wave solely rotating within its z-plane and seeding the longitudinal Ca^2+^ waves. Remarkably, this spatially restricted perpetual Ca^2+^ release is able to entrain all RyRs and make the cell insensitive to electrical pacing.

**Figure 7:**
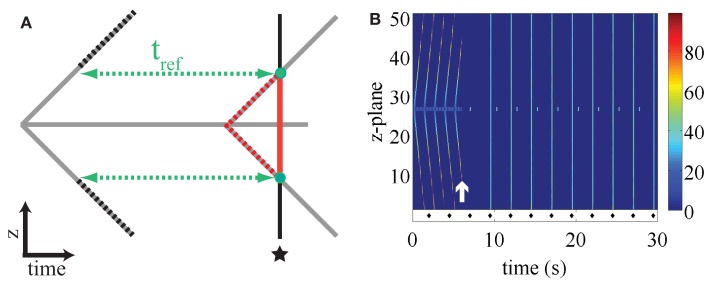
**(A)** Cartoon illustrating the ineffectiveness of pacing to stop ping waves. The ping wave corresponds to the horizontal gray line, while the longitudinal wave is denoted by the diagonal gray lines. The pacing occurs at the time indicated by the asterisk. See text for details. **(B)** Paced ping wave initiated as in Figure 4 where the refractory period is doubled at *t *= 6 s (white arrow). Each stimulus is indicated by a diamond symbol, the first stimulus occurs at *t *= 2 s. The initial refractory period in the central z-plane is 0.34 s, while *t*_ref_ = 1.2 s in the rest of the cell. Warm colors indicate substantial Ca^2+^ release site activity, while cool colors correspond to lesser Ca^2+^ release activity. Other parameter values as in Figure 2 and σ = 8.5 μM μm^3^/s, *t*_p_ = 2.5 s, initial fraction 0.8.

The mechanism by which electrical pacing fails to elicit a response once a ping wave has given rise to a fully developed longitudinal Ca^2+^ wave is illustrated in Figure 7A. It shows a cartoon of a ping wave (gray solid horizontal line) and the longitudinal Ca^2+^ wave (gray solid diagonal lines). At the time indicated by the asterisk, peripheral release sites are stimulated as would be the case for electrical pacing. At this point, the longitudinal Ca^2+^ wave has reached the z-planes marked by the two green dots. The RyRs that lie between the central z-plane and the currently responding z-planes (red solid line) cannot liberate Ca^2+^ because they are still refractory due to the actual longitudinal Ca^2+^ wave (dashed red lines). The RyRs that are located above and below the green dots have not yet recovered from refractoriness induced by the previous longitudinal Ca^2+^ wave (dashed black lines). The only channels that are ready to respond are those in the two z-planes indicated by the green dotes. However, such localized activity does not significantly perturb the Ca^2+^ flow of the longitudinal Ca^2+^ wave, hence rendering the external stimulation ineffective.

The results presented above indicate the robustness of ping waves in the face of electrical pacing. A key question is how such pro-arrhythmic activity could be quashed once initiated. Once again, the refractory period plays a critical role. Altering the refractory period can affect the sustainability of ping waves and their ability to trigger longitudinal Ca^2+^ waves. If the refractory period is too long, then ping waves will die out. The simple reason is that the Ca^2+^ concentration will decline substantially once a ping wave has traversed its z-plane so that there is an insufficient trigger to initiate another event. An example of the significance of the refractory period in altering ping wave activity is depicted in Figure 7B. At the beginning of the plot ping waves and their consequent longitudinal Ca^2+^ waves are evident. Since the longitudinal Ca^2+^ waves have reached the top and bottom of the cell, electrical pacing is ineffective. At the time indicated by the white arrow, the refractory period of the RyRs within the whole cell is immediately doubled. This provokes a rapid cessation of Ca^2+^ release and a quiescent phase whilst the RyRs recover. Since all triggers for autonomous Ca^2+^ release are halted, once the RyRs recover from being refractory the cell has to start responding to subsequent pacing events, and remain in-phase for the duration of stimulation. Some localized Ca^2+^ release activity is observed in the central z-plane, but is not sufficient to trigger a ping wave or longitudinal Ca^2+^ wave. These simulated data show that ping waves are critically dependent on refractory period and can be reversed once initiated.

## 4 Discussion

Fidelity of EC-coupling requires the appropriate translation of electrical stimulation into temporally- and spatially-coordinated Ca^2+^ signals. Any condition that allows Ca^2+^ dynamics to operate independently of electrical pacing has the potential to be arrhythmogenic. In particular, it is widely accepted that the activation of spontaneous Ca^2+^ signals can lead to arrhythmias by activating electrogenic ion transporters, or altering the responsiveness of cells to pacing. However, the triggers for spontaneous Ca^2+^ signaling, and the spatial pattern of such events are not well known.

In the present study, we investigated proarrhythmogenic Ca^2+^ signals in atrial myocytes. These cells have a well-defined geometry, and the relative positions of Ca^2+^ release sites (RyR clusters) are known (Mackenzie et al., 2004; Chen-Izu et al., 2006; Thul et al., 2012). Whilst confocal imaging has been used successfully by us and others to characterize Ca^2+^ signals within beating atrial myocytes (Sheehan and Blatter, 2003; Mackenzie et al., 2004) the physical limitations of such technology prevent the visualization of Ca^2+^ movement with the 3-dimensional volume of a cell in real time. We therefore implemented a mathematical model that realistically describes Ca^2+^ movement within an atrial myocyte (Thul et al., 2012). This model recapitulates the centripetal Ca^2+^ waves observed during electrical pacing, in addition to other features of atrial myocyte biology such as the response to positive inotropes. Moreover, the model has a predictive capacity in that it can demonstrate the genesis and sustainability of novel spontaneous Ca^2+^ signals, and their impact on physiological pacing.

When exploring the movement of Ca^2+^ under conditions of relatively low Ca^2+^ release, akin to the situation during heart failure, the model identified a novel type of self-supporting Ca^2+^ wave activity that we termed ping waves. These events are manifest as two counter-rotating Ca^2+^ waves that traverse a single z-plane of an atrial myocyte (Figure 3). Ping waves repeat themselves perpetually within the same z-plane. Our analyses suggests that ping waves are an inevitable consequence of the geometry of atrial myocytes. The re-triggering of ping waves is due to the Ca^2+^ concentration being continually above the threshold for RyR activation. Essentially, a new ping wave is triggered by the embers of the previous ping wave. From this, it is obvious that factors affecting cytosolic Ca^2+^ concentration (e.g., SERCA activity, release strength) and RyR activity (e.g., refractoriness) will impact on the potential triggering of successive ping waves. In particular, SERCA activity will serve to diminish the residual Ca^2+^ concentration that remains in the wake of a ping wave, so that a successive ping wave will not occur. In the present study, we altered the refractory period as a way of changing the time available for Ca^2+^ to be diminished by SERCA. As we demonstrate, short refractory periods favor ping wave activity, while they are eradicated by increasing refractory period. However, it is not the refractoriness of RyRs *per se* that controls ping wave firing, but rather the processes that occur after Ca^2+^ release termination. Long refractory periods quash ping wave activity because Ca^2+^ diffusion and SERCA activity remove the trigger.

Ping waves depend on the activation of just a few RyR clusters within a z-plane. This is necessary to generate a local Ca^2+^ signal that then propagates in a saltatory manner between the RyR clusters within the z-disk via successive rounds of diffusion and CICR. In the example shown in Figure 3, a ping wave was activated by triggering 6 peripheral RyR clusters. However, our simulations have shown that there is no need for any particular orientation or number of Ca^2+^ release sites to be involved (data not shown) – there simply needs to be a subcellular triggering event of sufficient magnitude to cause CICR in a localized spreading fashion.

A key aspect of ping wave survival is that the refractory period of the RyRs must be shorter than the recovery time of the cytosolic Ca^2+^ signal (Figure 7B). Otherwise there will be no residual Ca^2+^ to trigger another ping wave after the previous one has propagated and declined. We therefore consider that ping waves are most likely to occur under conditions such as those found in heart failure where the RyRs are believed to have increased leakiness, SR Ca^2+^ load is reduced, diastolic Ca^2+^ levels may be elevated and Ca^2+^ pumps are less effective at removing cytosolic Ca^2+^. These conditions would favor the scenario where RyRs emerge from being refractory in the presence of elevated Ca^2+^ concentration, with the consequent triggering of spontaneous Ca^2+^ release. There is no need for back flux of Ca^2+^ from adjacent z-planes to maintain ping wave activity, as the results for long refractory periods illustrate (Figure 5). Moreover, the existence of longitudinal Ca^2+^ waves in the presence of long refractory periods outside the central z-plane (Figure 5) demonstrates that the continuous Ca^2+^ signal generated by the ping wave is the main mechanism that triggers longitudinal Ca^2+^ waves under these conditions. For short refractory periods, ping waves are responsible for seeding the first longitudinal Ca^2+^ wave. All subsequent longitudinal Ca^2+^ waves occur autonomously due to the short refractory period similar to the results shown in Figure 2B.

The physiological processes underlying RyR refractoriness are not fully established, but are believed to involve increased cytosolic Ca^2+^, intrinsic channel gating properties and/or decreased SR Ca^2+^ loading. Current evidence suggests that SR Ca^2+^ load may be a principle determinant of RyR refractoriness, but is not the sole factor. Indeed, the SR can refill with Ca^2+^, but RyRs can still be refractory (Ramay et al., 2011; Belevych et al., 2012). In the present study, we deliberately avoided being descriptive about the mechanism underlying RyR refractoriness since the relative contributions of different processes that impinge on RyR activity are not known. Rather, we simply blocked RyR activity for a fixed time to convolve the effects of terminating Ca^2+^ release by cytosolic Ca^2+^, intrinsic gating or SR Ca^2+^ reduction. Essentially, with each mechanism, there would be a period of Ca^2+^ release followed by RyR closure, during which cytosolic Ca^2+^ can decline. Experimental data show that local Ca^2+^ release events recover within 200–250 ms, which is well within the period of ping waves described in this study (Figure 3). Hence, there is sufficient time between the end of one ping wave and the start of the next for SR Ca^2+^ to replenish, and RyR refractoriness to reverse.

An important feature of ping waves is their robustness, and their ability to make cells insensitive to electrical pacing. By themselves, ping waves would compromise atrial myocyte function because they occupy a fraction of the RyR clusters in a futile cycling of Ca^2+^ that is independent of pacing. More serious, however, is their ability to cause longitudinal Ca^2+^ waves by triggering CICR from neighboring z-planes. As demonstrated in Figure 6, if the refractory period of the cellular RyRs is less than the pacing frequency (as is normally the case in physiology), then the longitudinal Ca^2+^ waves will have an increasingly dominant effect on the cell. This is because ping waves can trigger longitudinal Ca^2+^ waves as soon as the RyRs emerge from being refractory. Whereas, the centripetal Ca^2+^ waves that are caused by pacing occur at longer intervals, thereby giving the longitudinal Ca^2+^ waves the chance to propagate. Once the longitudinal Ca^2+^ waves have reached the top and bottom of the cell centripetal Ca^2+^ waves cannot occur. Under these conditions, pacing an atrial myocyte would have no effect in terms of contraction. It is worth noting that persistent Ca^2+^ wave activity that is independent of electrical pacing has been observed in atrial myocytes when the cells were stimulated with endothelin-1 (Bootman et al., 2007).

Our simulation framework is based on the core components of intracellular Ca^2+^ dynamics: cytosolic Ca^2+^ diffusion, CICR trigger by cytosolic Ca^2+^, and Ca^2+^ extrusion by SERCA pumps. As such, the model is minimal, but it allows us to study in detail the Ca^2+^ dynamics in an atrial myocyte at the subcellular and cellular level at the same time. The low computational demand of the model comes at the price of some limitations. At the moment, we assume a static SR, which prevents us from incorporating SR depletion and refilling. Undoubtedly, SR Ca^2+^ dynamics is vital in shaping cardiac Ca^2+^ patterns (Thul et al., 2008; Zima et al., 2008), but it is worth noting that changing the release strength σ in the current model may be understood as a proxy to investigate, e.g., alterations of the total SR Ca^2+^ concentration. We here focused on deterministic Ca^2+^ release, where Ca^2+^ liberation starts at a fixed value of the cytosolic Ca^2+^ concentration and lasts for a given amount of time comparable to the duration of a Ca^2+^ spark. The main motivation for neglecting stochastic channel openings was our goal to better understand autonomous Ca^2+^ release in general and ping waves in particular. As we demonstrate, autonomous Ca^2+^ release can be achieved without spontaneous Ca^2+^ liberation by balancing release strength, SERCA pump activity and the refractory period. Introducing random openings of RyR cluster would certainly modify the shape of ping waves, but would not completely abolish them. As we showed previously (Thul et al., 2012) fluctuating Ca^2+^ release activity can be easily incorporated by randomizing the threshold for Ca^2+^ liberation. By construction, parameters such as the release strength σ or the release duration *t*_rel_ subsume a considerable number of mechanistic processes. Instead of modeling detailed molecular interactions, we chose a coarse-grained approach to investigate the consequences of changes at the micro scale – irrespective of their origin – for physiological and pathological Ca^2+^ signals.

Arrhythmias induced by overloading the SR are well documented. Our computational studies have highlighted another form of arrhythmogenic Ca^2+^ release that is favored by conditions of low SR Ca^2+^ load. Once acquired, these ping waves are difficult to stop and can persist indefinitely. Furthermore, although the simulations presented in this study have employed a ping wave occurring in a single z-plane, it is plausible that multiple ping waves could be present within a single cell. Analogous to cells acquiring multiple mutations of their DNA, several z-planes within an atrial myocyte could initiate ping wave phenomena and thereby trigger complex patterns of longitudinal Ca^2+^ wave formation. If the conditions described above are encountered then the progressive development of ping waves is inevitable.

## Conflict of Interest Statement

The authors declare that the research was conducted in the absence of any commercial or financial relationships that could be construed as a potential conflict of interest.
